# An investigation into possible interactions in the vestibular system and the cochlea during electrical stimulation

**DOI:** 10.3389/fnins.2025.1612253

**Published:** 2025-05-20

**Authors:** David Lanthaler, Andreas Griessner, Viktor Steixner, Julie Corre, Maurizio Ranieri, Samuel Cavuscens, Gautier Grouvel, Nils Guinand, Clemens M. Zierhofer, Angélica Pérez Fornos

**Affiliations:** ^1^Department of Mechatronics, University of Innsbruck, Innsbruck, Austria; ^2^Division of Otorhinolaryngology Head and Neck Surgery, Geneva University Hospitals and University of Geneva, Geneva, Switzerland

**Keywords:** vestibular implant, cochlear implant, electrical stimulation, balance restoration, bilateral vestibulopathy

## Abstract

**Introduction:**

The vestibular system is crucial for balance, spatial orientation, and gaze stabilization. Bilateral vestibulopathy (BV) severely impairs these functions, often co-occurring with severe to profound hearing loss. Combined cochleo-vestibular implants have the potential to rehabilitate these dual sensory impairments. These investigational devices have been used by a small group of subjects with both severe hearing loss and bilateral vestibular loss. When electrically stimulating both the cochlea and the vestibular system, understanding electrical interactions is essential for the successful fitting and operation of these combined implants. The aim of this study was to investigate the influence of vestibular pulses on cochlear and vestibular perception, and vice versa.

**Methods:**

In our study, we conducted experiments on three recipients of cochleo-vestibular implants, measuring auditory and vestibular perceptual thresholds under controlled conditions. The study examined three interaction paradigms: vestibulo-cochlear, cochleo-vestibular, and vestibulo-vestibular interactions. A staircase procedure was used to determine perceptual thresholds to evaluate the impact of concurrent stimulation on each sensory system.

**Results:**

The results showed subject-specific interactions, with significant threshold shifts observed in some cases due to the concurrent stimulation of cochlear and vestibular electrodes. Vestibulo-vestibular interactions consistently led to increased thresholds, indicating electrical interference within the vestibular system. In contrast, vestibulo-cochlear and cochleo-vestibular interactions demonstrated more variable effects, with threshold shifts observed in two of the three subjects.

**Discussion:**

These findings suggest that the dual stimulation of the cochlear and vestibular systems must be carefully managed to avoid compromising auditory or vestibular performance. In future research, focusing on larger cohorts could help to better understand the variability in subject responses. In addition, exploring functional effects of these interactions on subjects’ performances during normal implant use would complement the threshold measurements of the current study.

## 1 Introduction

The vestibular system in the inner ear is an essential contributor to the sense of balance and is also crucial for spatial orientation and gaze stabilization. A severe, bilateral impairment of the function of the vestibular system affects these abilities and is referred to as bilateral vestibulopathy (BV). BV is characterized by severe and debilitating symptoms such as chronic imbalance and visual difficulties in dynamic conditions. Because of the close anatomical proximity between the cochlea and the vestibular systems, pathological processes of the inner ear may also affect both sensory functions. Indeed, individuals suffering from profound hearing loss are the most susceptible to be affected also by vestibular disorders, with a prevalence of up to 50% (Handzel et al., 2006; Pajor and Jozefowicz-Korczynska, 2007; Wiener-Vacher, 2008; Jacot et al., 2009; Cushing et al., 2013; Batuecas-Caletrio et al., 2015; Verbecque et al., 2017; Nayak et al., 2022). Rehabilitation with combined cochleo-vestibular implants is currently being investigated and could help to overcome both sensorineural losses to a certain degree (Guyot et al., 2016; Phillips et al., 2020; Miguel et al., 2024; Vermorken et al., 2024).

Due to the anatomical proximity of the two end-organs, the possibility of achieving simultaneous stimulation of the cochlear and vestibular branches of the VIIIth cranial nerve without adverse effects needs to be carefully investigated as it could result in spread of activation adversely impacting auditory and/or vestibular performance. Concrete hints to this effect have been found in previous studies by our group, where some subjects have reported auditory percepts upon electrical stimulation delivered via the vestibular electrodes [see, e.g., (Guinand et al., 2015)]. Understanding this potential electrical interaction between the different stimulation electrodes is therefore essential for the successful fitting and operation of combined cochleo-vestibular implants.

To our knowledge, there are only two studies available on the topic to date (Phillips et al., 2020; Miguel et al., 2024). Miguel et al. investigated whether electrical stimulation from a cochlear implant could also stimulate the otolith end-organ of the vestibular system and improve balance, as a practical alternative to direct vestibular stimulation. They analyzed cross talk between the cochlea and the otolith organ in 4 subjects using trans-impedance matrix analysis as well as a speech comprehension test (Disyllabic Word Test) and the Dynamic Gait Index. No effective cross-stimulation could be found, and they concluded that effective otolith stimulation can only be achieved via direct electrical stimulation of the otolith end-organs (Miguel et al., 2024). Philips et al. conducted a study to quantify auditory and vestibular interactions during interleaved stimulation with a combined cochleo-vestibular prosthesis in 3 subjects. They found no auditory sensation when stimulating the vestibular system alone and no sensation of motion or slow-phase eye movement from cochlear stimulation alone. However, they found significant interactions during stimulation with a combined vestibular and cochlear prosthesis resulting in changes of pitch and loudness in the auditory part as well as changes in slow-phase eye velocities (Phillips et al., 2020).

In both referenced studies, electrode designs and placements and implant models are used which are substantially different to the ones we use in our study. These differences in setup lead to results not being directly comparable to possible interactions found in our design. We also aim to study possible interactions between simple electrode pulse trains at a very fundamental level, while the other two studies deal with more functional interactions.

The main objective in our study was to investigate potential interactions when using combined stimulation of one cochlear and one vestibular electrode as well as two vestibular electrodes. For this, we conducted an experiment utilizing a combined cochleo-vestibular implant, where we determined and compared the vestibular and auditory perceptual thresholds for single electrodes under controlled conditions for combined stimulations. We hypothesized that in our configuration audio-vestibular interactions would be observed as threshold shifts, which we aimed to quantify.

## 2 Methods

### 2.1 Implant and subjects

The cochleo-vestibular implant is a modified cochlear implant providing 3 electrode branches to be implanted in the ampulla of each semicircular canal and an intracochlear array with 9 electrode contacts. Device and implantation surgery details have been described previously (Fornos et al., 2014; Guinand et al., 2015).

The current case study was conducted on 3 recipients of a combined cochleo-vestibular implant who received their devices from the University Hospital Geneva. Currently such combined implants are used for vestibular stimulation in research only settings and thus the number of recipients is very limited world-wide. This explains the low number of patients included in this study. The demographic information of the participants is shown in Table 1.

**Table 1:** Subject demographics.

Subject	S1	S2	S3
Sex	F	F	F
Implantation age	66	72	67
Implantation year	2021	2021	2013
Etiology	DFNA9	Meningitis	Trauma
Implant side	R	L	L

### 2.2 Hardware and software

The setup was controlled by a desktop computer running custom-made research software (Matlab R2017b, The Mathworks Inc., Natick, Massachusetts, United States). The computer communicated this information to the implanted stimulator via the manufacturer’s MAX programming interface (MED-EL, Innsbruck, Austria) and the cochlear implant system’s speech processor—a SONNET 2 (MED-EL, Innsbruck, Austria). The testing procedures were generated and analysed using custom-made research software (Matlab R2020a, The Mathworks Inc., Natick, Massachusetts, United States).

### 2.3 Interaction paradigms

As mentioned above, the aim of this study is to investigate the influence of vestibular stimulation on both auditory and vestibular perception thresholds and vice versa. The main outcome measure was the current threshold (i.e., lowest current eliciting an auditory or vestibular percept) applied on a probe electrode (that could be a vestibular or a cochlear electrode) with or without concurrent presentation of another electrical signal applied on a perturbation electrode (vestibular or cochlear). The three possibilities for probe and perturbation electrodes used in this study are summarized as the interaction conditions shown in Table 2 and can be described as follows.

(A) Influence of vestibular stimulation on auditory perception thresholds (vestibulo-cochlear interactions), characterized using the probe signal on a cochlear electrode and the perturbation signal on a vestibular electrode.
(B) Influence of cochlear stimulation on vestibular perception thresholds (cochleo-vestibular interactions), investigated with the probe signal on a vestibular electrode and the perturbation signal on a cochlear electrode.
(C) Vestibulo-vestibular interactions, investigated with the probe signal on a vestibular electrode and the perturbation signal on another vestibular electrode.

**Table 2:** Interaction conditions for the experiment defining the targeted end-organ.

Condition	A	B	C
Probe	Cochlear	Vestibular	Vestibular
Perturbation	Vestibular	Cochlear	Vestibular

Cochleo-cochlear interactions were not examined here, as they have already been thoroughly investigated in previous studies (Boëx et al., 2003).

### 2.4 Electrode selection

All active vestibular electrodes were used in each subject and vestibulo-vestibular interactions (interaction condition C) were evaluated with all available vestibular electrode pairs. To investigate cochleo-vestibular interactions, the geometric distance between electrodes was determined with the help of computer tomography scans. The geometrically closest cochlear electrode to each vestibular electrode was chosen for each combination (shown in Table 3). The listed electrode combinations per subject were used for the perturbation and probe signals depending on the tested paradigm defined in the following section.

**Table 3:** Combinations of the geometrically closest neighbouring electrodes per subject.

Subject	Vestibular electrode	Cochlear electrode
S1	LAN	E2
	SAN	E2
	PAN	E9
S2^a^	LAN	E2
	PAN	E8
S3^b^	LAN	E4
	SAN	E5

PAN = posterior ampullary nerve, LAN = lateral ampullary nerve, SAN = superior ampullary nerve.

a Subject S2 had facial nerve activation on the SAN electrode at very low current levels and was thus not evaluated in this study.

b In subject S3, the PAN electrode did not evoke perception or vestibular-ocular responses and was thus not included.

### 2.5 Electrode characterization

The first step in this study involved the determination of the dynamic range (DR) of each individual electrode. The DR is defined as the current range between the threshold (lowest perceivable level) and the upper comfortable level (UCL) and was determined by the subject’s feedback using a visual analog scale. For each individual electrode, subjects were presented with pulse trains with the durations used in the actual experiments. The stimulation amplitude was increased step by step until the subject detected the signal for the first time (i.e., threshold level). Then, the signal was gradually increased until the upper comfortable level (UCL) was reached. At each stimulation amplitude the intensity of percepts was quantified using a visual analog scale of 10 levels (0 meaning no perception, 1 meaning very weak, up to 10 meaning very strong). Note that in these experiments UCL could be determined based on a very strong percept reported by the subject but also in case of unintentional activation of nearby structures such as the facial nerve. The DR of an individual electrode is influenced by both the pulse phase duration and the burst duration. As these durations are different for vestibular and cochlear electrodes used as probe or perturbation signal, determining the DR required testing all possible combinations of durations across all electrodes.

### 2.6 Perceptual threshold assessments

**Figure 1:**
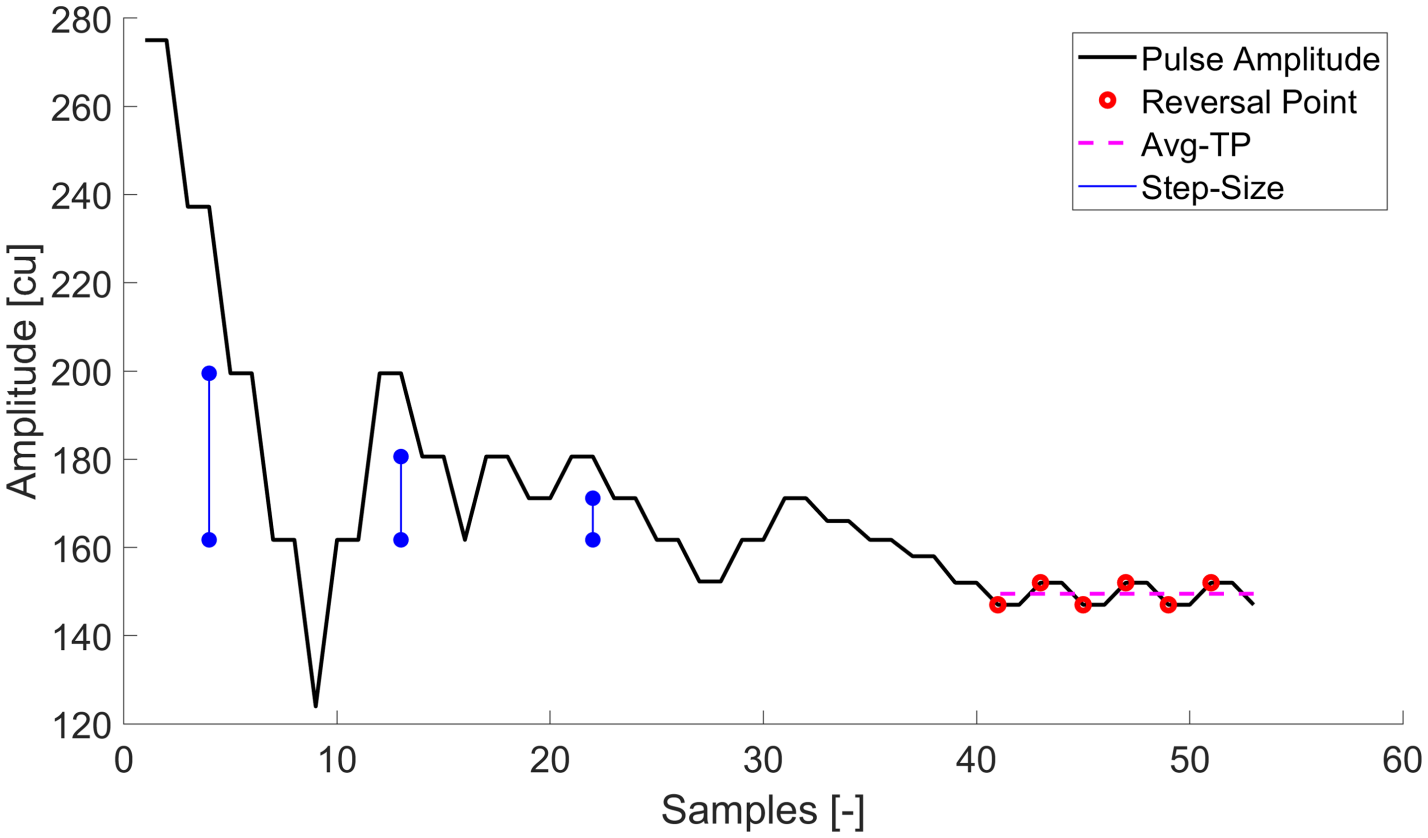
Example of the 3-AFC staircase procedure. The amplitude represents the tested amplitude level for the probe signal. The step-size by which the amplitude levels were increased or decreased were halved 3 times after every second reversal. The last six reversals points mark the values used to determine the average threshold, which is illustrated by the Avg-TP (red dashed line) and used as the main outcome measure.

Once the DR for each individual electrode was determined, we proceeded to the measurement of the threshold levels in each of the different conditions. We used an adaptive staircase psychophysical procedure (Levitt, 1971; Boëx et al., 2003). In such a procedure, the amplitude level of the probe threshold is decreased after two consecutive correct trials (i.e., stimulus detected) and for every incorrect response (i.e., stimulus undetected) it is increased by one step-size. This staircase procedure, illustrated in Figure 1, converges to a detection rate of approximately 70.7% (Levitt, 1971) and has already been used on previous investigations for cochlear threshold comparisons (Boëx et al., 2003).

The step-size was halved after every second reversal point from an initial step-size of 37.76 current units (cu) down to a minimum step size of 4.72 cu. As the protocol is demanding for the patient, the bigger initial step-size allowed convergence on the first reversal as efficiently as possible. The minimum adjustable step-size was limited by the current resolution of the device within the used amplitude range of all subjects. Each staircase ended when six reversal points with the minimum step-size were recorded (a compromise between length of the staircase run for the subject and a converged threshold).

**Figure 2:**
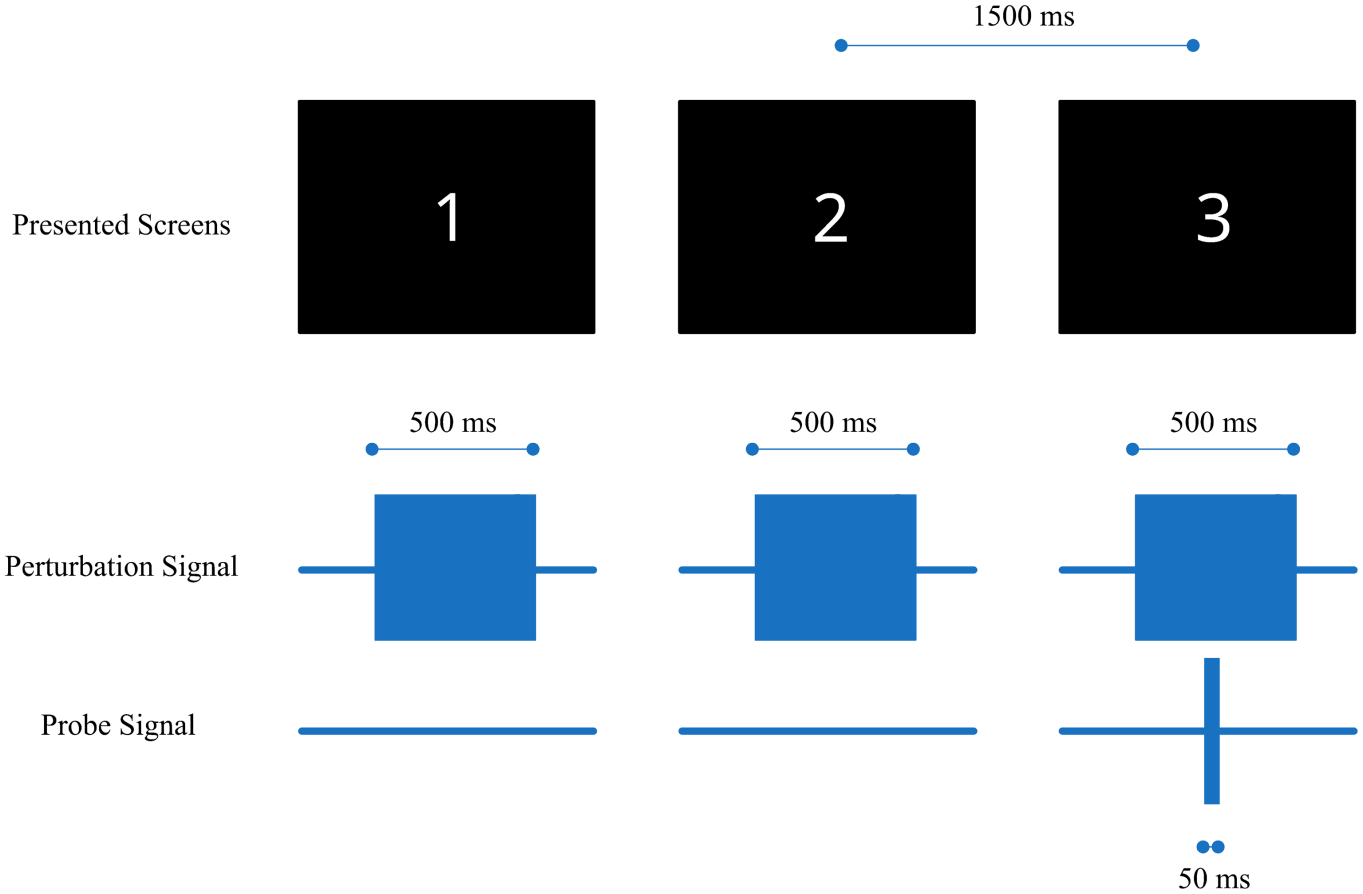
Illustration of the stimulation sequence, consisting of three 500 ms perturbation burst signals (middle row) consecutively delivered to one electrode and a 50 ms probe burst signal (bottom row) delivered to another electrode during only one of the 3 presentations (third stimulus in the current example). The numbered screens (top) were presented to the subject in the dark room during each stimulus presentation.

Each experimental trial consisted of three consecutive presentations of a “perturbation” burst lasting 500 ms. During one of these three presentations, a “probe” signal (50 ms duration) was also presented, as illustrated in Figure 2. For the interaction conditions (see Section 2.3 and Table 2) the perturbation signal level was set to 50% of the DR measured during individual electrode characterization (see Section 2.5). We also performed “reference” measurements where the amplitude level of the perturbation signal was set zero (i.e., no perturbation signal). The starting amplitude for the probe signal was set to ensure that the subjects could easily identify it at the beginning of the measurements, corresponding to a perceived level of 50% of the DR.

The position of the probe signal was randomized within each trial sequence and across subjects. Each consecutive presentation was indicated to the subject via a numbered screen 1 to 3 in a dark room with an interval of 1.5 s between each stimulus. After the three presentations, the subject had to report which presentation was perceived “different” compared to the other two. If the reported number matched the presentation screen which included both the perturbation and the probe signal, the trial was valued as correct, otherwise as incorrect. This procedure is known as a three alternative forced choice (3-AFC) method (Levitt, 1971; Boëx et al., 2003).

**Figure 3:**
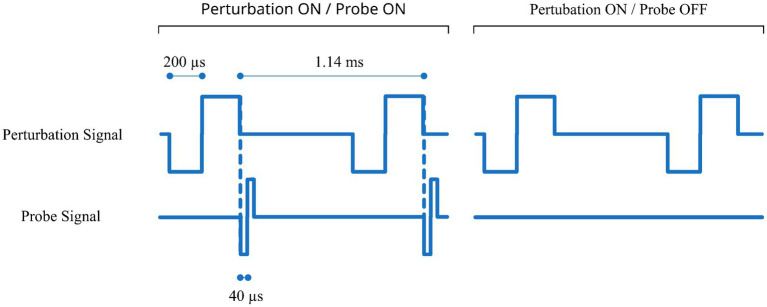
Detailed pulse timing for the probe and perturbation signal for two cases with and without probe signal (left and right panel, respectively). In this example the probe signal is on a cochlear electrode and the perturbation signal is on a vestibular electrode.

The pulse bursts of the probe and the perturbation signals in the lower half of Figure 2 are composed of individual pulses with the specific timing shown in Figure 3. In screens including the probe signal the individual probe pulses follow immediately after the perturbation pulses. The pulse phase durations and pulse rate were chosen to best represent real-world usage of a cochleo-vestibular implant, aligning them as close as possible to the ones from cochlea implant users in their daily life and remaining consistent with those used in a previous study (Lanthaler et al., 2021). The phase durations for the cochlea and the vestibular electrode pulses were 40 μs and 200 μs, respectively, with a rate of 878 pps and an inter-phase gap of 2.1 μs.

### 2.7 Statistical analyses

For each condition (A–C), thresholds of seven perturbed electrodes were compared pairwise to their corresponding unperturbed reference thresholds. Paired t-tests were applied for conditions B and C, where the Shapiro–Wilk test on normality passed (*p* = 0.709 and *p* = 0.877) while for condition A, a Wilcoxon signed-rank test was used, as the data did not follow a normal distribution (*p* < 0.05).

## 3 Results

**Figure 4:**
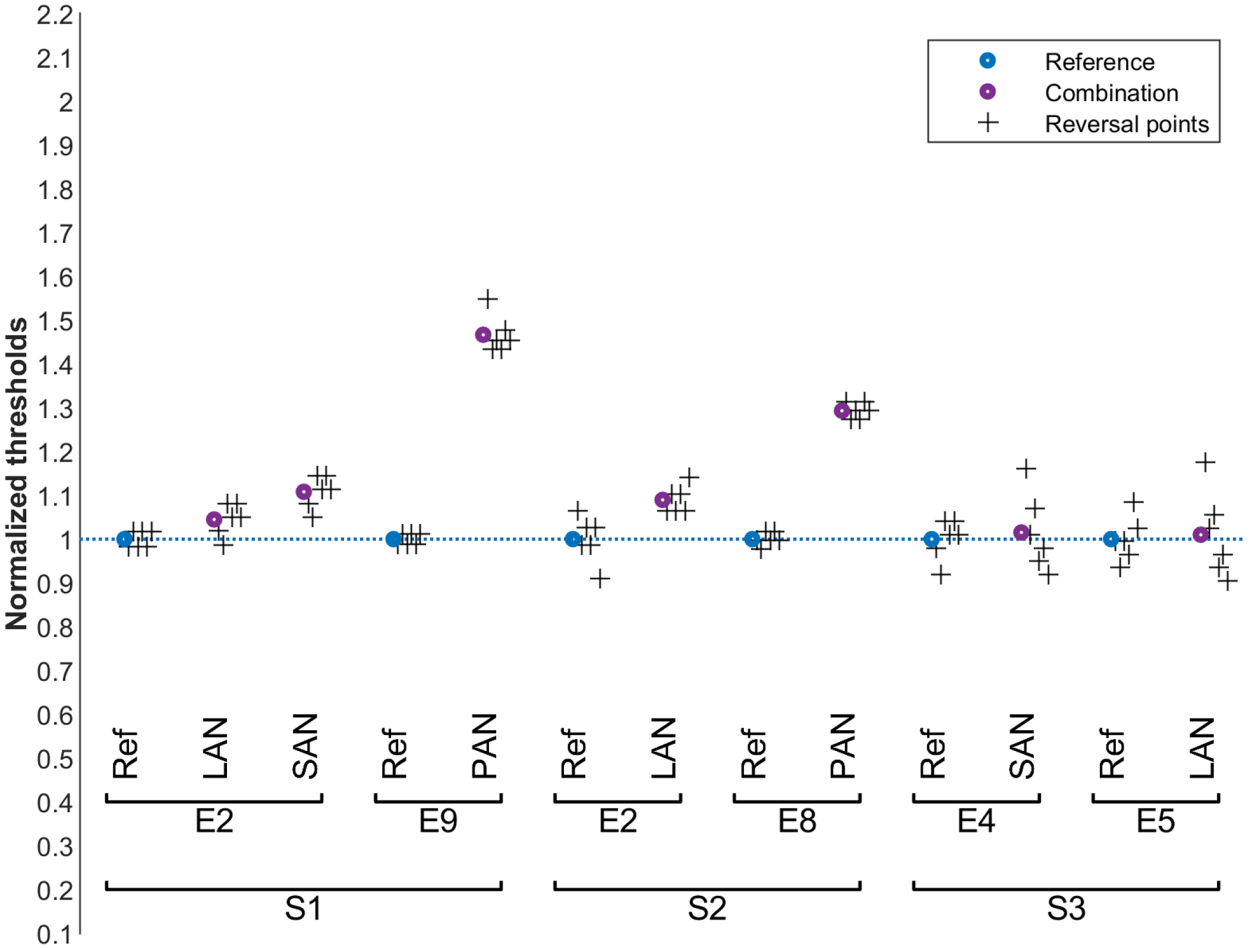
Cochlear thresholds in the presence of vestibular perturbation signals (condition A). Blue: normalized reference thresholds measured on the probe electrode alone (without perturbation signal). Purple: normalized threshold values upon simultaneous stimulation of the perturbation electrode. The final six reversal points of each run are marked with plus symbols. The cochlear probe electrodes are named below the upper brackets and the vestibular perturbation electrodes above.

**Figure 5:**
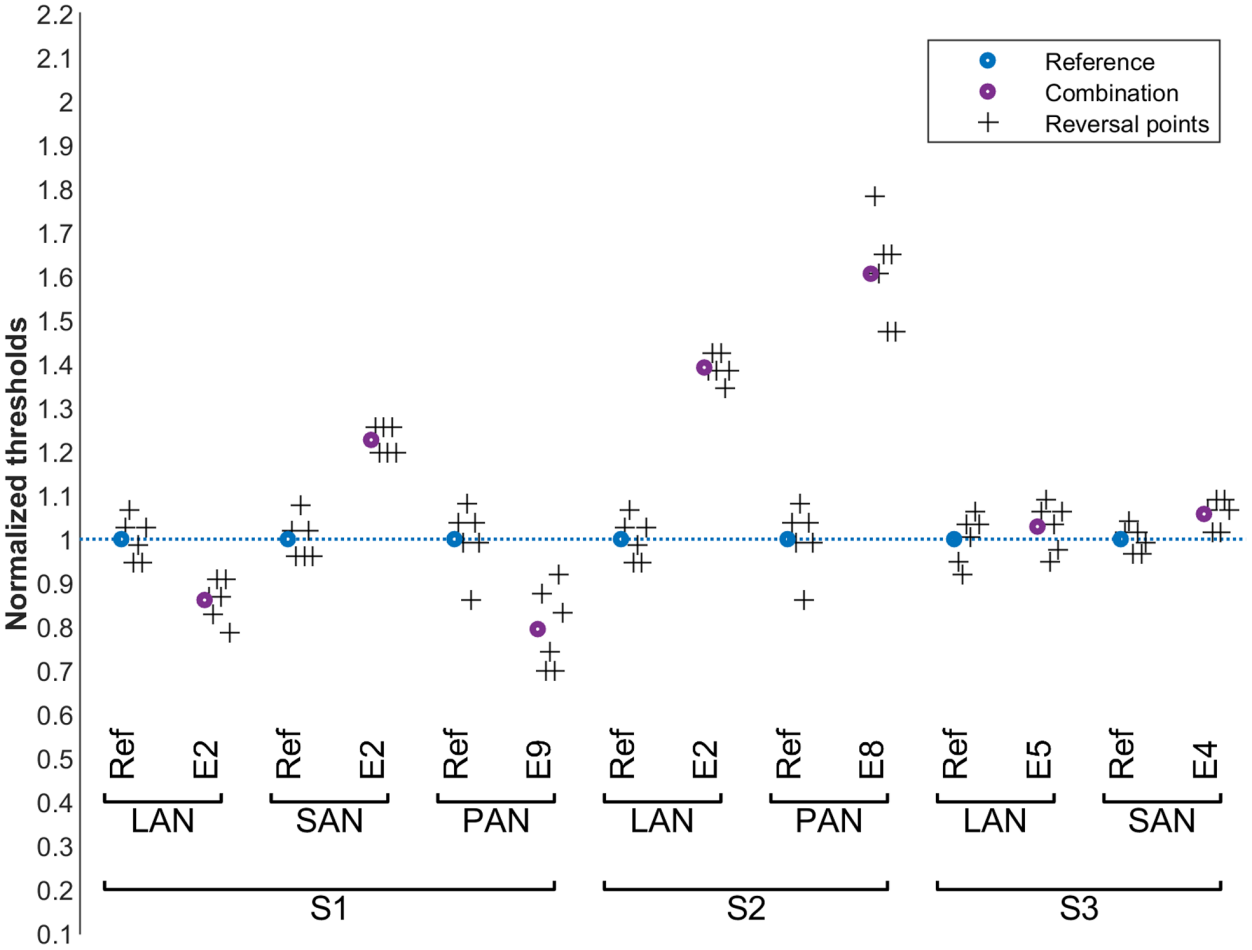
Vestibular perception thresholds in the presence of cochlear perturbation signals (condition B). Blue: normalized reference thresholds measured on the probe electrode alone (without stimulating the perturbation electrode). Purple: normalized threshold values upon simultaneous stimulation of the perturbation electrode. The final six reversal points of each run are marked with plus symbols. The vestibular probe electrodes are indicated below the upper brackets and the cochlear perturbation electrodes above.

**Figure 6:**
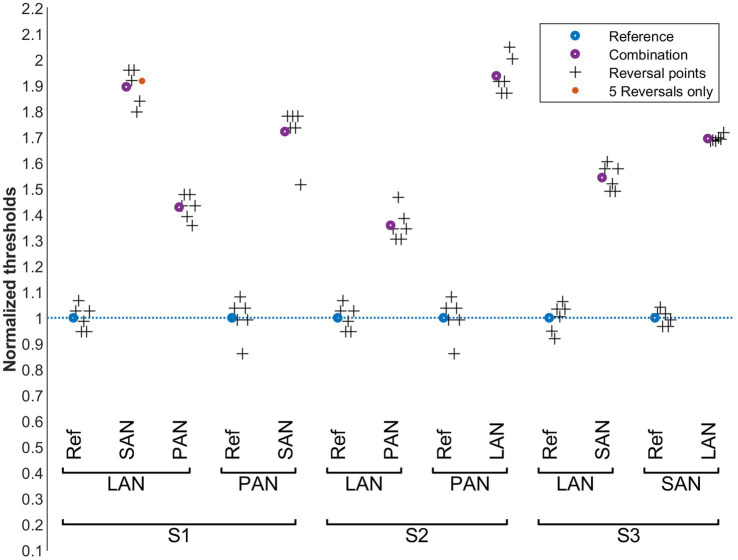
Vestibular perception thresholds in the presence of another vestibular perturbation signals (condition C). Blue: normalized reference thresholds measured on the probe electrode alone (without stimulating the perturbation electrode). Purple: normalized threshold values upon simultaneous stimulation of the perturbation electrode. The final six reversal points of each run are marked with plus symbols. There is one case with only 5 reversals which is marked with a red circle. The vestibular probe electrodes are indicated below the upper brackets and the vestibular perturbation electrodes above.

The results for the individual subject thresholds in each condition are presented in Figures 4–6. Results were normalized with respect to the reference measurements performed without perturbation for every probe electrode. In general, the mean values were calculated from the last six reversal points recorded with the smallest step-size within each staircase recording and are indicated in the figures. The evaluation of vestibulo-vestibular interactions was particularly challenging for the participants. Therefore, in this case one measurement that had less than six reversals is marked with an asterisk and the number of reversals is provided.

The influence of vestibular stimulation on auditory thresholds, condition A, is presented in Figure 4. For S1 and S2, auditory thresholds slightly increased (< 10%) with the presence of vestibular perturbations in the LAN electrode. This was also the case for the SAN electrode for S1. For these same patients, interactions were stronger when the perturbation was applied to the PAN electrode (30% to 45%). This was not the case for S3, where the auditory thresholds remained unchanged despite the vestibular perturbation (for this subject the thresholds did not change for any of the tested electrode pairs). Pairwise comparisons of all reference values with the corresponding perturbed values showed a significant increase in thresholds (Wilcoxon signed rank test, *p* = 0.016).

The influence of cochlear stimulation on vestibular perception thresholds (condition B) is shown in Figure 5. As in condition A, the thresholds in subject S3 seem to be relatively insensitive to the perturbation signals. For S1, vestibular perception thresholds decreased by approximately 20% upon cochlear perturbations for probe electrodes LAN and PAN, while they increased by a similar proportion when the probe electrode was the SAN electrode. For S2, the vestibular perception thresholds increased by 40% to 60% upon cochlear perturbations for both the LAN and PAN electrodes. A pairwise comparison of all reference values with the corresponding perturbed values did not show a significant overall increase or decrease of the threshold values (paired t-test, *p* = 0.246).

Purely vestibular interactions were evaluated in condition C. The results are shown in Figure 6. Substantial interactions were observed: measured thresholds markedly increased by about 40% to 90% for all subjects and in all electrode combinations tested. A pairwise comparison of all reference values with the corresponding perturbed values showed a significant increase of the thresholds (paired t-test, *p* < 0.001).

## 4 Discussion

The primary objective of this study was to investigate potential interactions between electrodes in the cochlea and electrodes in the semicircular canals of the vestibular system during combined stimulation. In our experiment, we compared the perception thresholds of individual probe electrodes with the thresholds of these same electrodes while a perturbation signal was applied to a different electrode. If significant interactions occurred between these electrodes, a change in the threshold of the probe electrode would be expected in the presence of the perturbation. This could, on one hand, lead to a decrease in the threshold, as the probe and perturbation stimulation could sum up if the same nerve fibers are stimulated. On the other hand, an interaction might make it more difficult for participants to detect the threshold of a probe signal when an additional interfering perturbation signal is present. Another possibility would be that thresholds increase due to forward masking, i.e., to the adaptation of a nerve to the masking perturbation signal when the probe signal occurs.

In conditions A and B, where the probe and perturbation electrodes were positioned in different end-organs in the inner ear (anatomically close to each other), results from subject S3 showed little to no difference in the mean threshold with and without a perturbation signal. For S1 and S2, thresholds increased for all electrodes in condition A with the largest increase for the PAN electrode. For condition B, thresholds obtained with the perturbation were also noticeably different from the reference measurements. From these results, the hypothesis that there are electrical or neurological interactions between the end-organs cannot be rejected. Such interactions appear to cause a deterioration in auditory thresholds in the presence of vestibular perturbation (condition A). When assessing vestibular thresholds under cochlear perturbation (condition B), a difference in the presence of the perturbation signal was consistently observed. However, in some cases an increase of threshold was observed and in other cases the threshold decreased. This could mean that while in some cases the probe and perturbation stimulation signals summate (the same nerve fibers are stimulated or a central facilitation is activated) in other cases we observe “forward masking.” This result should however be interpreted with caution, since determining vestibular thresholds is a particularly challenging task for participants.

In condition C, thresholds consistently increased across subjects when vestibular electrodes were used both for the probe and for the perturbation signal. This indicates electrical interactions likely due to the proximity of the stimulated electrodes within the same end-organ.

### 4.1 Limitations and future work

This exploratory study has shown that there appear to be subject-dependent interactions. Future research could benefit from examining whether these interactions consistently emerge in larger cohorts. Additionally, it would be valuable to explore whether the observed interactions can be linked to specific pathologies.

To investigate potential interactions between the two inner ear end-organs, we only selected one cochlear electrode that was geometrically closest to the corresponding vestibular electrode being tested. This choice was made in order to allow enough time to explore all possible combinations within the inner ear in conditions that maximized the probability of observing potential interactions, while still limiting the experimental time to make it possible to patients to complete the protocol in a realistic time frame. Yet, in the future it would also be interesting to further examine different vestibular and cochlear electrode combinations.

An interesting aspect for future research is the temporal alignment of the perturbation and probe signal pulses. This might be particularly relevant when considering scenarios where the pulses overlap, as in parallel stimulation strategies already used in cochlear implants. Parallel stimulation would offer the advantage of achieving higher overall stimulation rates for both end-organs, especially since vestibular pulse durations typically significantly exceed cochlear pulse durations.

Finally, beyond the psychophysical threshold measurements conducted in this study, an important future direction would be to investigate the “functional” effects of these interactions on subjects’ performance during normal implant use. This could include dynamic visual acuity tests performed while hearing a perturbation sound, or speech comprehension tests conducted during vestibular stimulation.

## 5 Conclusion

In this study, we investigated the influence of vestibular pulses on cochlear and vestibular perception and vice versa. For single electrode stimulation in both the cochlea and the vestibular system, subject specific interactions were found. While no discernable change in threshold was found for one subject, we observed clear interactions for the other two subjects in our experimental configuration. When vestibular electrodes were used for both the probe and the perturbation signal, interactions leading to an increase in the detectable threshold current levels were observed in all cases.

The variability of the overall results motivates future studies to better understand how interactions might be influenced by different parameters, e.g., electrode positions, anatomical differences, or even physio-pathological processes (progression/evolution of disease in the different etiologies) and their real functional impact in the overall rehabilitation strategy.

## Data Availability

The datasets presented in this study can be found in online repositories. The names of the repository/repositories and accession number(s) can be found at: https://osf.io/s9u6z/?view_only=3d1d28134cd34206b51e8b7b79ff838f.
